# The Way towards Ultrafast Growth of Single‐Crystal Graphene on Copper

**DOI:** 10.1002/advs.201700087

**Published:** 2017-05-30

**Authors:** Zhihong Zhang, Xiaozhi Xu, Lu Qiu, Shaoxin Wang, Tianwei Wu, Feng Ding, Hailin Peng, Kaihui Liu

**Affiliations:** ^1^ State Key Laboratory for Mesoscopic Physics School of Physics Collaborative Innovation Center of Quantum Matter Peking University Beijing 100871 China; ^2^ Academy for Advanced Interdisciplinary Studies Peking University Beijing 100871 China; ^3^ School of Materials Science and Engineering Ulsan National Institute of Science and Technology (UNIST) Ulsan 689–798 Republic of Korea; ^4^ Centre for Nanochemistry College of Chemistry and Molecular Engineering Beijing Science and Engineering Center for Nanocarbons Peking University Beijing 100871 China

**Keywords:** Cu foil, CVD, single crystal, ultrafast graphene growth

## Abstract

The exceptional properties of graphene make it a promising candidate in the development of next‐generation electronic, optoelectronic, photonic and photovoltaic devices. A holy grail in graphene research is the synthesis of large‐sized single‐crystal graphene, in which the absence of grain boundaries guarantees its excellent intrinsic properties and high performance in the devices. Nowadays, most attention has been drawn to the suppression of nucleation density by using low feeding gas during the growth process to allow only one nucleus to grow with enough space. However, because the nucleation is a random event and new nuclei are likely to form in the very long growth process, it is difficult to achieve industrial‐level wafer‐scale or beyond (e.g. 30 cm in diameter) single‐crystal graphene. Another possible way to obtain large single‐crystal graphene is to realize ultrafast growth, where once a nucleus forms, it grows up so quickly before new nuclei form. Therefore ultrafast growth provides a new direction for the synthesis of large single‐crystal graphene, and is also of great significance to realize large‐scale production of graphene films (fast growth is more time‐efficient and cost‐effective), which is likely to accelerate various graphene applications in industry.

## 1 Introduction

**Figure 1:**
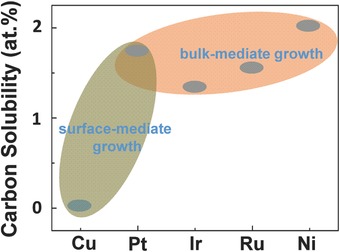
The solubility of carbon in several transition metals at 1000 °C.^76^

Graphene, a two‐dimensional material with carbon atoms bonded in a honeycomb lattice, has attracted immerse interests in the past decade due to its exceptional properties and various applications.^1^, ^2^, ^3^, ^4^, ^5^, ^6^, ^7^, ^8^, ^9^, ^10^, ^11^, ^12^ The growth of large‐area high‐quality graphene films is fundamental for the upcoming graphene applications. Chemical vapour deposition (CVD) method offers good prospects to produce large‐size graphene films due to its simplicity, controllability and cost‐efficiency.^13^, ^14^, ^15^, ^16^, ^17^, ^18^, ^19^, ^20^, ^21^, ^22^, ^23^, ^24^, ^25^, ^26^, ^27^, ^28^, ^29^, ^30^, ^31^, ^32^, ^33^, ^34^, ^35^, ^36^, ^37^, ^38^, ^39^, ^40^, ^41^, ^42^, ^43^, ^44^, ^45^, ^46^, ^47^, ^48^, ^49^, ^50^, ^51^, ^52^, ^53^, ^54^, ^55^, ^56^, ^57^, ^58^, ^59^, ^60^, ^61^, ^62^, ^63^, ^64^, ^65^, ^66^, ^67^, ^68^, ^69^, ^70^, ^71^, ^72^, ^73^, ^74^, ^75^ Many researches have verified that graphene can be catalytically grown on metallic substrates, like ruthenium (Ru),^13^, ^14^ iridium (Ir),^15^, ^16^ platinum (Pt),^17^, ^18^, ^19^, ^20^ nickel (Ni)^21^, ^22^, ^23^ and copper (Cu)^30^, ^31^, ^32^, ^33^, ^34^, ^35^, ^36^, ^37^, ^38^, ^39^, ^40^, ^41^, ^42^, ^43^, ^44^, ^45^, ^46^, ^47^, ^48^, ^49^, ^50^, ^51^, ^52^, ^53^, ^54^, ^55^, ^56^, ^57^, ^58^, ^59^, ^60^, ^61^, ^62^, ^63^, ^64^, ^65^, ^66^, ^67^, ^68^, ^69^, ^70^, ^71^, ^72^, ^73^ et al., and also can be directly grown on insulating substrates, such as SiC,^24^, ^25^ SiO_2,_ 26, 27 sapphire,^28^ and h‐BN.^29^, ^30^ Considering that direct graphene growth on desired insulating substrate without transfer process is promising for integration into silicon‐based technologies, it would be a favourable way for industrial application of graphene. However, despite a lot of efforts, so far the synthesized graphene on insulating substrates is typically of poor quality, small domain size and low growth rate, as a result of that the direct growth relies on the thermal decomposition of the carbon resource. Up to now, many challenges remain in this direction and far‐away from the large‐area high‐quality graphene production. On the other hand, graphene grown on metallic substrates possesses higher quality and can reach large size easily, thus is a way close at hand for reliable graphene production to realize various applications. The carbon solubility in metals determine the graphene growth behaviour (see **Figure** **1**).^76^ Ru, Ir, Pt and Ni have high carbon solubility and their usage leads to a segregation growth process, in which carbon first dissolves into the bulk metals at a high temperature and segregates to form graphene after reaching the carbon saturation, and the graphene grows up with carpet growth mode. Ru and Ir are noble metals and economically unfavourable. Previous studies on graphene grown on Ru and Ir mainly focused on the surface science rather than graphene synthesis. Although, Pt is also expensive, the development of the bubbling transfer method makes the repeated growth on Pt available and greatly reduces cost.^18^ The high catalytic activity and high melting temperature of Pt enables the fast growth of graphene at high temperatures. Besides, the graphene growth on Pt can also be realized by a surface‐mediated process and millimetre‐scale single‐crystal graphene domains can be obtained.^19^ A smart surface engineering of polycrystalline Pt by coating with silicon‐containing film makes the graphene growth free from the effects of Pt substrate which improves the crystallinity, uniformity and the domain size of graphene.^20^ With this eutectic substrate the growth rate was increased to 120 µm min^−1^ at 1150 °C, more than an order of magnitude higher than typical reported ones. Pt is one of the promising substrates, while at the moment it still cannot meet the demands for the large‐scale synthesis of graphene. Ni and Cu are much cheaper and widely available substrates that make the industrialized production of graphene feasible. High solubility of carbon in Ni leads to the formation of multilayer graphene.^21^, ^22^ Achieving a uniform monolayer graphene on Ni is still a great challenge. Especially, Cu with low carbon solubility, serving as catalytic substrate, makes the continuous uniform high‐quality monolayer graphene films accessible.^31^, ^32^, ^33^, ^34^ Nowadays, the large‐scale production and transfer of graphene film from Cu foils has been realized by the roll‐to‐roll technique.^69^, ^70^ Thus, the CVD growth on Cu foils is believed to be the most promising method to realize the industrial production of single‐crystal graphene films.

However, as‐synthesized graphene films on Cu foils are typically polycrystalline and the grain boundaries degrade the properties greatly, limiting its high‐end applications.^77^, ^78^ To demolish all negative effects of grain boundaries, the growth of single‐crystal graphene is of great importance. Currently the most prevailing method to achieve large single‐crystal graphene is to suppress the nucleation density, growing the island as large as possible from a single nucleus. State‐of‐the‐art, 1.5 inch‐sized single‐crystal graphene has been achieved from one nucleus by a creative local feeding technique.^52^ However, it may be difficult to make the size even larger (for example 30 cm in diameter) because the nucleation is a random event and it is inevitable that new nucleus would form during the very long growth process, even with extremely low feeding gas. On the other hand, if the graphene growth rate is extremely high, once one nucleus forms, it grows up rapidly before new nuclei form, which provides a new route to achieve the growth of very large single‐crystal graphene.^36^ Additionally, ultrafast growth can make the growth process time‐ and energy‐efficient, contributing to the scale‐up production of graphene for coming large‐scale applications. Therefore, improving graphene growth rate in CVD process is a crucial and significant topic. In this review, we first discuss the merits of ultrafast growth and then investigate the factors to promote the growth rate. Finally, the ways towards ultrafast growth are summarized and new research activities and directions are proposed.

## 2 The Merits of Ultrafast Graphene Growth

**Figure 2:**
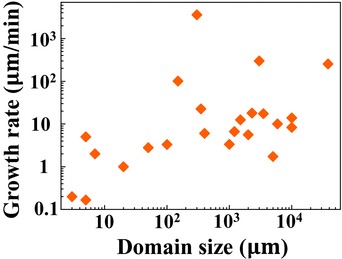
Graphene domain size versus growth rate on Cu foil in 2009–2016.^31^, ^32^, ^33^, ^34^, ^35^, ^36^, ^37^, ^38^, ^39^, ^40^, ^41^, ^42^, ^43^, ^44^, ^45^, ^46^, ^47^, ^48^, ^49^, ^50^, ^51^, ^52^, ^53^, ^54^

**Figure 3:**
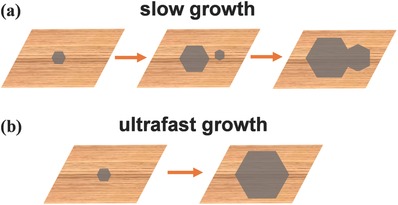
Schematics of graphene growth with (a) low and (b) ultrahigh growth rate.

After about a decade's efforts, the domain size of single‐crystal graphene has increased more than four orders of magnitude, from micrometres to inches (**Figure** **2**).^31^, ^32^, ^33^, ^34^, ^35^, ^36^, ^37^, ^38^, ^39^, ^40^, ^41^, ^42^, ^43^, ^44^, ^45^, ^46^, ^47^, ^48^, ^49^, ^50^, ^51^, ^52^, ^53^, ^54^ On the other hand, before 2016, the growth rate has not improved as much, varying in the range of several µm min^−1^. That means to grow 12‐inch single‐crystal graphene films typically needs almost one day. This time‐ and energy‐consuming process is not suitable for the large‐scale production. Only from 2016, several breakthroughs made the growth rate exceed 100 µm min^−1^,^45^, ^46^ even astonishingly up to 3600 µm min^−1^.^36^ Ultrafast growth can greatly shorten the process and its significance is far beyond this time or energy efficiency. It is known that the crystal nucleation is a random event, and thus new nuclei would inevitably form during the long growth process, as schematically illustrated in **Figure** **3**a. That's also one reason why the single‐crystal domain size is still limited to inch size so far, even with the most advanced growth methods. If the growth rate can be extremely high, the nucleus would grow up so rapidly that new nuclei do not form (Figure 3b). Therefore, the ultrafast growth, if possible, is ideal to synthesize large single‐crystal graphene domains. If the domains are unaligned, the fast growth enables the polycrystalline graphene films consisting of large domains with less grain boundaries and therefore higher quality. If the domains are aligned and seamless stitched together, such as in the epitaxial growth on Ge(100) or Cu(111) substrate, large single‐crystal graphene film can be produced in a short time.^58^, ^59^ All in all, improving the growth rate is a new and effective route, although its importance has not been paid too much attention earlier, to grow high‐quality or super‐large single‐crystal graphene films.

## 3 The Ways towards Ultrafast Graphene Growth

**Figure 4:**
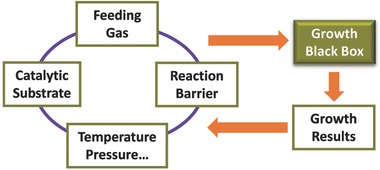
Schematic illustration of the way to improve the graphene growth.

Cu has very low carbon solubility and as a result, the graphene growth on Cu surface follows the surface‐mediated growth mode.^79^, ^80^, ^81^ The good thermal stability of CH_4_ makes it a preferred carbon source to prevent pyrolysis at high temperatures, which is favourable to surface‐mediate growth of high‐quality graphene.^33^ Generally speaking, the graphene CVD growth on Cu involves four processes:^82^ (i) absorption of CH_4_ onto Cu surfaces; (ii) catalytic dissociation of CH_4_ by Cu, resulting in C species CH_x_ (x = 0–3); (iii) surface diffusion of C species; (iv) C attachment onto graphene domain edges. A large number of experiments have shown that these four processes in graphene growth are dominated by the catalytic substrate, feeding gas, reaction barriers, temperature and pressure (**Figure** **4**). So to make proper control over these conditions is essential to realize ultrafast growth of graphene.

### 3.1 Control the Catalytic Substrate

The metallic substrate in the CVD graphene growth also serves as catalyst for the CH_4_ decomposition. As the source for graphene formation is mainly from the active carbon species (CH_x_, x = 0–3) that is catalytically decomposed from CH_4_ and absorbed on the Cu surface, increasing active carbon on Cu surface can directly enhance the graphene growth rate. However, the high carbon species concentration on the Cu surface also leads to more nucleation and thus smaller graphene domain sizes. People desire large graphene domains and try to have little active carbons on Cu surface to suppress the nucleation density with the compromise of growth rate. That is the reason why the reported growth rate is usually very low on Cu. It seems to be a dilemma in optimizing both the domain size and growth rate. A good way to get out of this dilemma is to use Cu‐Ni alloy substrate. Ni has higher catalytic activity and carbon solubility than Cu and can be alloyed with Cu with any element content, thus changing the growth behaviors.^52^, ^83^ Partial carbons species first dissolve into the Cu‐Ni alloy bulk and then segregate out during the graphene growth. By controlling the Ni content, the proportion of dissolved carbon can be tuned and faster graphene growth can be realized by more carbon supply from both surface diffusion and bulk segregation.^52^ Another way is to pre‐oxidize Cu foil or heat it in a non‐reducing atmosphere to make a small concentration of O remain on the surface or in the bulk of Cu foil when growing graphene, suppressing the nucleation and improving the graphene growth rate.^64^, ^84^

Besides the composition, the configuration of substrate also affects the growth rate distinctly. When rolling or vertically stacking Cu foils via direct physical contact with a gap of about tens of micrometres, the gas flow in the narrow spaces of the stacked Cu foils could be in the molecular flow regime, where the CH_4_ would have high colliding frequency and substantially enhance the local concentration of carbon flux, and the hence graphene growth rate is improved.^46^, ^71^ Also the special substrate configurations were designed which formed confining spaces to suppress the loss of Cu by evaporation and redeposition and thus led to a smoother surface for fast graphene growth, especially under low pressure.^46^, ^54^

Also the surface properties, including surface index, step, bunches, impurities and defects, have dramatic influence on the graphene growth. Generally, Cu foils used to grow graphene are polycrystalline and on different facet surfaces graphene growth behaviours are different. The growth rate on Cu(111) is higher than (100) and other high index facets, attributed to higher diffusion and improved absorption of hydrocarbon precursor.^85^ Besides, Cu(111) has three‐fold symmetry, fitting the lattice symmetry of graphene and very small lattice mismatch with graphene. The orientation of graphene epitaxially grown on (111) surface is consistent with underlying copper lattice.^59^, ^60^ Therefore the single‐crystal Cu(111) is an ideal substrate for high‐quality graphene growth. The inhomogeneities on copper substrate surface are often considered as nucleation sites for carbon‐containing species. It was found that the growth rate decreases rapidly when growth fronts of neighbouring sheets approach each other when the CVD is conducted under low temperature or with low hydrocarbon feeding.^40^, ^55^ The in situ observation by environmental scanning electron microscope (ESEM) showed there is obvious mutual influence between neighbouring graphene domains during the growth.^86^ The isolated nucleus grows much faster than the one surrounded by the other nuclei. So the high nucleation density inevitably results to the overall low growth rate. Besides, the impurities or step bunches can distort the growth trajectory and thus lower the growth rate. In addition, the inhomogeneities influence the growth rate slightly by affecting the diffusion process.^53^, ^54^, ^55^, ^56^, ^57^ Proper surface treatment technique, like long‐time annealing,^65^ polishing,^56^, ^68^, ^87^ melting and resolidification,^72^, ^73^, ^74^ and the above mentioned special Cu stacking configuration can accelerate the graphene growth by minimizing surface roughness.

### 3.2 Control the Feeding Gas

CH_4_ is typically used as the carbon source for graphene growth and thus can affect the growth rate directly. Apparently a higher concentration of CH_4_ would lead to faster growth of graphene over the Cu surface, while it also results in higher nucleation density. The nucleation occurs when the C species on the active sites are oversaturated and if the CH_4_ flow is too low that there will be no nucleation and growth.^38^, ^39^ Scientists utilize multi‐stage CH_4_ feeding, namely low feeding at the nucleation stage and high feeding in the growth stage, to achieve lower nucleation density but large graphene domain size.^45^, ^64^

H_2_ also plays vital roles in the graphene growth process. Actually, Cu can catalyse hydrogen dissociation reaction as well.^88^ Higher partial pressure of H_2_ would reduce surface sites, leaving fewer available Cu sites for CH_x_ (x = 1–4) dissociative chemisorption. In addition, the dissociated H atoms are active to etch the edge carbon atom of existing graphene domains.^41^, ^89^ Although H_2_ is impeditive to the rapid growth, it is essential to achieve monolayer growth.

**Figure 5:**
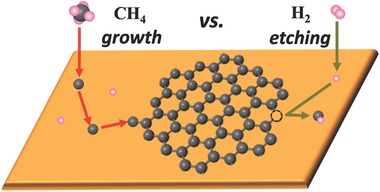
The graphene growth by attachment of dissociated C onto graphene domain edge versus the H_2_ etching by detachment of C from graphene domain edge.

As discussed above, growth process is a balance between carbon diffusion/deposition and hydrogen etching (schematically illustrated in **Figure** **5**), thus an optimization of the ratio would improve the CVD growth.^62^ Methane/hydrogen ratio of the growth also affects nucleation density or domain size, an optimization of the ratio may produce graphene films with good quality and fast growth rate.^33^

### 3.3 Control the Reaction Barriers

In the growth process, the dissociation of CH_4_, namely, CH_x_ $\overset{\;\mathrm{Cu}\;}{\to }$ CH_x–1_ + H (x = 4, 3, 2, 1), is thermodynamically unfavourable and the reaction barrier is relative high.^66^ Alternatively, the partial dehydrogenated carbon (CH_x–1_) may combine with each other and finally form H‐terminated graphene domains. Thus further C species edge attachment and lattice incorporation for the graphene domain growing up require dehydrogenation. In a typical graphene growth process, the growth rate can be either diffusion‐limited (insufficient active C supply) or edge‐attachment‐limited.

**Figure 6:**
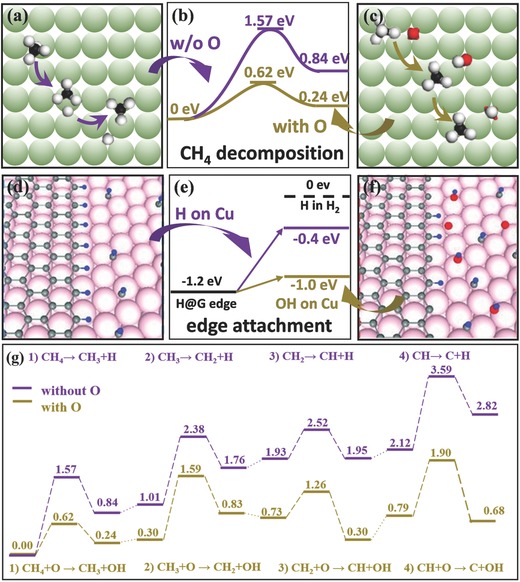
a–c) Schematic illustration and Density‐Functional‐Theory (DFT) calculated energy barrier for the CH4 dissociation with and without oxygen on Cu surface. Reproduced with permission.^36^ 2016, Nature Publishing Group. d–f) Atomic‐scale schematics of graphene edge structure on Cu surface with and without oxygen and DFT calculated energies of different configurations of H. Reproduced with permission.^35^ 2013, American Association for the Advancement of Science. g) Summary of Climbing Image‐Nudged Elastic Band (CI‐NEB) calculations of CH4 full dissociation reactions with and without oxygen on Cu surface. The energy barriers of the decomposition reactions with oxygen (golden line), are 0.62, 1.29, 0.53 and 1.11 eV, respectively; the energy barriers of reactions without oxygen (purple line) are 1.57, 1.37, 0.59, and 1.47 eV, respectively.

It was reported the O on Cu surface can reduce the energy barrier to the dissociation of CH_4_ via the reaction CH_x_ + O $\overset{\;\mathrm{Cu}\;}{\to }$ CH_x–1_ + OH (x = 4, 3, 2, 1). As shown in **Figure** **6**a–c, during the first step of the CH_4_ decomposition, the O atom on the Cu surface is able to interact with the H atom by the formation of O–H bond^36^ and, therefore, the energy barrier of the elementary reaction is greatly reduced by 0.95 eV (Figure 6b). This significant barrier reduction, according to the theories of reaction kinetics, was then estimated to increase the rate of the decomposition reaction and the concentration of CH_3_ radicals by several orders of magnitude. Considering that a CH_4_ molecule decomposition is a complicated process, we carefully evaluated the full dehydrogenation of CH_4_ by Climbing Image‐Nudged Elastic Band (CI‐NEB) calculation and the result is presented in Figure 6g. It clearly shows that, for each elementary reaction, the existence of an O atom can greatly reduce both the reaction barrier and the reaction energy. The results presented above confirm that the existence of oxygen on the Cu surface will greatly promote the rate of decomposition and therefore the concentration of various active carbon species, such as CH_3_, CH_2_, CH and C, on the metal surface and/or in the gas phase. Thus, no matter which kind of C species should be the key participant of the graphene growth, a quick supply of C source around each graphene domain is guaranteed.

Also, given that the H on the Cu has lower energy in the form of OH compared with that of H on Cu^35^ (Figure 6d–f), the reaction barrier reduction also leads to lower activation energy of edge dehydrogenation. That means the edge‐attachment can be easier in the presence of O on Cu. Therefore, the O absorbed on Cu can enhance both the active C supply and the edge‐attachment activity. Thus, the graphene growth rate can be improved in an exponential way according to the Arrhenius equation.^90^ Besides, oxygen can passivate the active sites on the Cu surface and thus suppress the nucleation density.^35^, ^36^ Experiment results showed that a continuous O supply in the growth process can improve the growth rate by 2–4 orders of magnitude.^36^

### 3.4 Control the Temperature and Pressure

As an active factor in the CVD growth of graphene, temperature can affect the growth rate significantly. The catalytic activity of Cu is lower compared to other mentioned metal catalyst and CH_4_ is chemically stable. Therefore, the temperature must be much higher using Cu as substrate and CH_4_ as carbon source. Usually, 1000 °C or above is applied to achieve the growth of high‐quality single‐crystal graphene. At lower temperature, like 800 °C, graphene can be synthesized but with poor quality.^91^ Higher temperature can accelerate the thermally activated processes, dissociation of CH_4_, diffusion of active carbon and attachment of C species onto graphene nuclei. Also, higher temperature can decrease the nucleation density of graphene.^32^, ^48^, ^92^ As the temperature is close to the melting temperature of Cu, the pre‐melting surface provides locally an atomically flat and electronically homogeneous support for graphene growth with a minimum interference from the support. Also the absence of surface inhomogeneities leads to a reduced nucleation density.^86^ It was also demonstrated that the high‐quality graphene can also be grown on liquid copper with very high growth rate.^73^ On the other hand, there were experiments revealing that the sublimation of copper at the high temperatures will reduce the continuity of graphene domains and thus won't favour the growth of high‐quality graphene.^93^ The positive effects of high temperature on growth can be partial counteracted by the more active C species desorption at higher temperature.^40^, ^41^ Generally, at a lower temperature, small and dense graphene islands are formed, while large and sparse ones are favoured at higher temperatures.^33^

Pressure is another important factor in the growth process. It has been widely accepted that low pressure CVD can readily achieve uniform monolayer graphene film. At lower pressure, the lower gas flux results in fewer collisions and a higher diffusivity coefficient, and thus the absorption of CH_4_ on Cu surface is enhanced.^34^ It was also reported that the total pressure of the system have similar effects on the graphene growth with methane/hydrogen ratio.^94^ At very low pressure, the growth of graphene is slow due to insufficient carbon supply and the graphene domains can hardly merge into a complete film. With enough carbon supply onto Cu surface, the surface reaction regime is rate limiting, and the reaction rate is largely dependent upon temperature. Actually, the uniform monolayer graphene film can also be synthesized at atmospheric condition with low feeding of CH_4_.^34^, ^62^ When the CH_4_ flux is high, the self‐limited growth process would be broken and multilayer graphene may form.^34^, ^95^ Also the gas reaction may occur and produce amorphous carbon particulate, which may deposit on Cu surface and form active centre for the nucleation or damage the existing graphene. Therefore too high pressure or too much CH_4_ is not a good choice for the growing of high‐quality graphene.

Based on the above discussion, the effects of temperature and pressure on growth rate are interactive. Appropriate temperature and pressure conditions can be reached to produce graphene with high growth rate and good quality.^33^

## 4 Conclusion and Outlook

To improve the growth rate of CVD graphene is essential for both fundamental research and industrial applications. In this review, the available ways towards the ultrafast graphene growth are discussed in details. Proper catalytic substrate can achieve the higher growth rate, while it is more powerful in the control of layer number. The optimization of feeding gas ratio has limited capacity to improve the grow rate. The deduction of reaction barrier, for example by the introduction of oxygen, can improve the growth rate exponentially and turns out to be a very powerful way to realize ultrafast growth.

In the future, further work is required for faster growth and larger graphene single crystals. Several possible routes are proposed: (i) to explore a more efficient way to reduce the reaction barrier for the graphene growth; (ii) to epitaxially grow well aligned graphene domains and make them seamlessly stitch together into a complete single‐crystal film; (iii) to combine with roll‐to‐roll technique to realize streamline production. Route (i) can be realized by effective introduction of oxidative substance like O onto Cu surface, to enhance the dissociation of hydrocarbon. This is expected to readily obtain millimetre‐scale single‐crystal graphene islands in several minutes. Actually, route (ii) has been implemented by growing graphene on Ge(110)^58^ or Cu(111).^59^, ^60^ However, the single‐crystal graphene film has limited size (wafer scale) due to the limited single‐crystal substrate size and the quality is less pleasant due to the not perfect alignment of individual graphene islands. Therefore, to achieve large‐area high‐quality graphene film, preparation of large‐area substrate and improvement of graphene alignment are vital and critical issues in the future works. Once these two issues settled down, the size of single‐crystal can only be limited by the size of furnace and it can be foreseen that the production of metre‐scale single‐crystal graphene film is at the corner. This is very important for large‐scale graphene industrial applications and also opens a new research field for the growth of other 2D materials to use graphene as supporting substrate. Besides, new powerful technique to realize real‐time observation of the growth process, like newly designed CVD furnace, in situ electron microscopy, should be developed to get a deeper insight of the growth mechanism.

## Conflict of Interest

The authors declare no conflict of interest.
